# A female patient with retinoblastoma and severe intellectual disability carrying an X;13 balanced translocation without rearrangement in the *RB1* gene: a case report

**DOI:** 10.1186/s12920-019-0640-2

**Published:** 2019-12-05

**Authors:** Makiko Tsutsumi, Hiroyoshi Hattori, Nobuhiro Akita, Naoko Maeda, Toshinobu Kubota, Keizo Horibe, Naoko Fujita, Miki Kawai, Yasuko Shinkai, Maki Kato, Takema Kato, Rie Kawamura, Fumihiko Suzuki, Hiroki Kurahashi

**Affiliations:** 1 Division of Molecular Genetics, Institute for Comprehensive Medical Science, Fujita Health University, 1-98 Dengakugakubo, Kutsukake-cho, Toyoake, Aichi 470-1192 Japan; 2 Department of Clinical Genetics, National Hospital Organization, Nagoya Medical Center, Nagoya, Japan; 3 Department of Pediatrics, National Hospital Organization, Nagoya Medical Center, Nagoya, Japan; 4 Department of Ophthalmology, National Hospital Organization, Nagoya Medical Center, Nagoya, Japan; 5 Genome and Transcriptome Analysis Center, Fujita Health University, Toyoake, Japan; 6 Center for Collaboration in Research and Education, Fujita Health University, Toyoake, Japan

**Keywords:** Retinoblastoma, Balanced X-A translocation, X-inactivation

## Abstract

**Background:**

Female carriers of a balanced X; autosome translocation generally undergo selective inactivation of the normal X chromosome. This is because inactivation of critical genes within the autosomal region of the derivative translocation chromosome would compromise cellular function. We here report a female patient with bilateral retinoblastoma and a severe intellectual disability who carries a reciprocal X-autosomal translocation.

**Case presentation:**

Cytogenetic and molecular analyses, a HUMARA (Human androgen receptor) assay, and methylation specific PCR (MSP) and bisulfite sequencing were performed using peripheral blood samples from the patient. The patient’s karyotype was 46,X,t(X;13)(q28;q14.1) by G-banding analysis. Further cytogenetic analysis located the entire *RB1* gene and its regulatory region on der(X) with no translocation disruption. The X-inactivation pattern in the peripheral blood was highly skewed but not completely selected. MSP and deep sequencing of bisulfite-treated DNA revealed that an extensive 13q region, including the *RB1* promoter, was unusually methylated in a subset of cells.

**Conclusions:**

The der(X) region harboring the *RB1* gene was inactivated in a subset of somatic cells, including the retinal cells, in the patient subject which acted as the first hit in the development of her retinoblastoma. In addition, the patient’s intellectual disability may be attributable to the inactivation of the der(X), leading to a 13q deletion syndrome-like phenotype, or to an active X-linked gene on der (13) leading to Xq28 functional disomy.

## Background

Balanced translocations generally have no impact on the clinical phenotype of the carrier unless the breakpoint disrupts a dosage sensitive gene. However, X; autosome (X-A) translocations in females are more complex because of the X-chromosome inactivation (XCI), which is a mechanism of dosage compensation of X-linked genes between females and males [1, 2]. Since the derivative chromosome of an X-A translocation harboring the X-inactivation center may be subject to inactivation, its autosomal region is subject to unfavorable inactivation. This results in cellular dysfunction due to inactivation of critical genes leading to the pathological change or death of cells. In consequence, cells in females carrying an X-A translocation generally undergo selective inactivation of the normal X chromosome.

Retinoblastoma (RB, OMIM #180200) is a malignant intraocular tumor occurring in young children, which is caused by mutations in both alleles of the *RB1* gene [3]. Individuals with heterozygous germline pathogenic variations frequently develop bilateral retinoblastoma in infancy. Constitutional chromosomal abnormalities involving 13q14, where the *RB1* gene is located, are found in a subset of cases with a predisposition for RB. Large deletions that include the *RB1* gene lead to widely variable clinical phenotypes, including intellectual disability, referred to as 13q deletion syndrome [4, 5]. We here describe a female patient with bilateral retinoblastoma and severe intellectual disability who was found to carry an X;13 translocation. Cytogenetic and molecular analysis revealed inactivation of der(X) and the *RB1* gene in a subset of her cells, which explains the cause of her phenotype.

### Case presentation

#### Cytogenetic analysis

Blood samples from the study subjects were obtained with informed consent in accordance with local institutional review board guidelines. An Epstein-Barr virus (EBV) transformed Lymphoblastoid cell line (LCL) was established from the peripheral blood derived from the patient as described previously [6]. Conventional G-banding and fluorescence in situ hybridization (FISH) analyses were performed using LCL. Cytogenetic analyses were performed using a standard method. The Zyto*Light* SPEC RB1/13q12 Dual Color Probe (ZytoVision GmbH, Bremerhaven, Germany) was used to detect the *RB1* gene. A bacterial artificial chromosome (BAC) DNA was labeled with SpectrumGreen or SpectrumOrange-labeled 2′-deoxyuridine-5′-triphosphate using the Nick-Translation Kit (Abbott Japan, Tokyo, Japan). To visualize late replicating regions, LCL was arrested with thymidine (300 μg/ml) for 18.5 h followed by a treatment with bromodeoxyuridine (BrdU; 25 μg/ml) for 6.5 h after release from the arrest. Metaphase cells were labeled with a FISH probe for the X chromosome centromere (Cytocell, Cambridge, UK), and BrdU was detected with Alexa Fluor 594-conjugated mouse anti-BrdU antibody (ThermoFisher Scientific, Tokyo, Japan).

#### HUMARA assay

For HUMARA assays, genomic DNA was extracted from peripheral blood or LCL using the QuickGene DNA whole blood DNA kit L (Kurabo, Osaka, Japan). Restriction enzyme treatment followed by PCR analysis was then conducted as described previously [7].

#### Methylation-specific PCR

Bisulfite conversion of genomic DNAs obtained from the peripheral bloods of the patient and healthy human volunteers was first performed with the Epitect Bisulfite kit (QIAGEN, Tokyo, Japan). PCR was then carried out using EpiTaq HS (Takara, Kusatsu, Japan). EpiScope Methylated HeLa gDNA (Takara) was used as a positive control. The primers used in these analyses were designed with the BiSearch software [8] and are listed in Table 1.

**Table 1:** Primers used for MSP in this study

Primer^*a*^	Forward (5′-3′)	Reverse (5′-3′)	Size (bp)
RB1-M	GGGAGTTTCGCGGACGTGAC	ACGTCGAAACACGCCCCG	163
RB1-U	GGGAGTTTTGTGGATGTGAT	ACATCAAAACACACCCCA	163
q13.1-M	AAAACCCGAACGCAACGAAC	TCGTCGTAGTTGTTATCGTC	120
q13.1-U	AAAACCCAAACACAACAAAC	TTGTTGTAGTTGTTATTGTT	120
q14.11-M	GCGCGATGGAGTTTTAGTAC	CGAAAAAAAACCCGAACGAC	214
q14.11-U	GTGTGATGGAGTTTTAGTAT	CAAAAAAAAACCCAAACAAC	214
q14.3-M	CCGCCTAACGTCAATAAAAC	GTGTTTAGAACGACGGGTGC	160
q14.3-U	CCACCTAACATCAATAAAAC	GTGTTTAGAATGATGGGTGT	160
q21.33-M	TAGGTTTCGTTTTTCGCGTTC	CTTTAACTCCCCGCTTCCGC	226
q21.33-U	TAGGTTTTGTTTTTTGTGTTT	CTTTAACTCCCCACTTCCAC	226
q31.1prox-M	AGATTCGGCGTTAGGTAGGGC	CGCGCTCTAAAAAATTAAAAC	368
q31.1prox-U	AGATTTGGTGTTAGGTAGGGT	CACACTCTAAAAAATTAAAAC	368
q31.1 dis-M	CGTACTACTACCCCCGCTAC	GCGTTTTTTAGCGTTTTTTA	194
q31.1 dis-U	CATACTACTACCCCCACTAC	GTGTTTTTTAGTGTTTTTTA	194
q31.2-M	GCCGCTACGCTAAAAAACGA	CGTATTTTTCGGTTTGGGTTCGC	283
q31.2-U	ACCACTACACTAAAAAACAA	TGTATTTTTTGGTTTGGGTTTGT	283
q31.3-M	ACGAAATACCTACGCGCCAAC	CGCGGGTTAATAAAGTTTAC	149
q31.3-U	ACAAAATACCTACACACCAAC	TGTGGGTTAATAAAGTTTAT	149
q32.3-M	CGCGACTCCGAACAATAACC	AATGTAGTTATAATCGCGGC	243
q32.3-U	CACAACTCCAAACAATAACC	AATGTAGTTATAATTGTGGT	243
q34-M	AGGTTATAGGTTAGACGCGGC	CGAAACGAACGAAAACTAAC	252
q34-U	AGGTTATAGGTTAGATGTGGT	CAAAACAAACAAAAACTAAC	252

^a^Given as the corresponding chromosomal band of the long arm of chromosome 13

#### Bisulfite sequencing

The *RB1* promoter region was amplified by PCR as described previously [9]. The PCR products were then used as the template for secondary PCR with primers containing sequencing adaptors. Amplicon sequencing was subsequently performed on an Illumina MiSeq in accordance with the manufacturer’s protocol to obtain paired-end 150 bp reads. Sequencing data were analyzed with Bismark software [10].

#### Patient characteristics

**Fig. 1:**
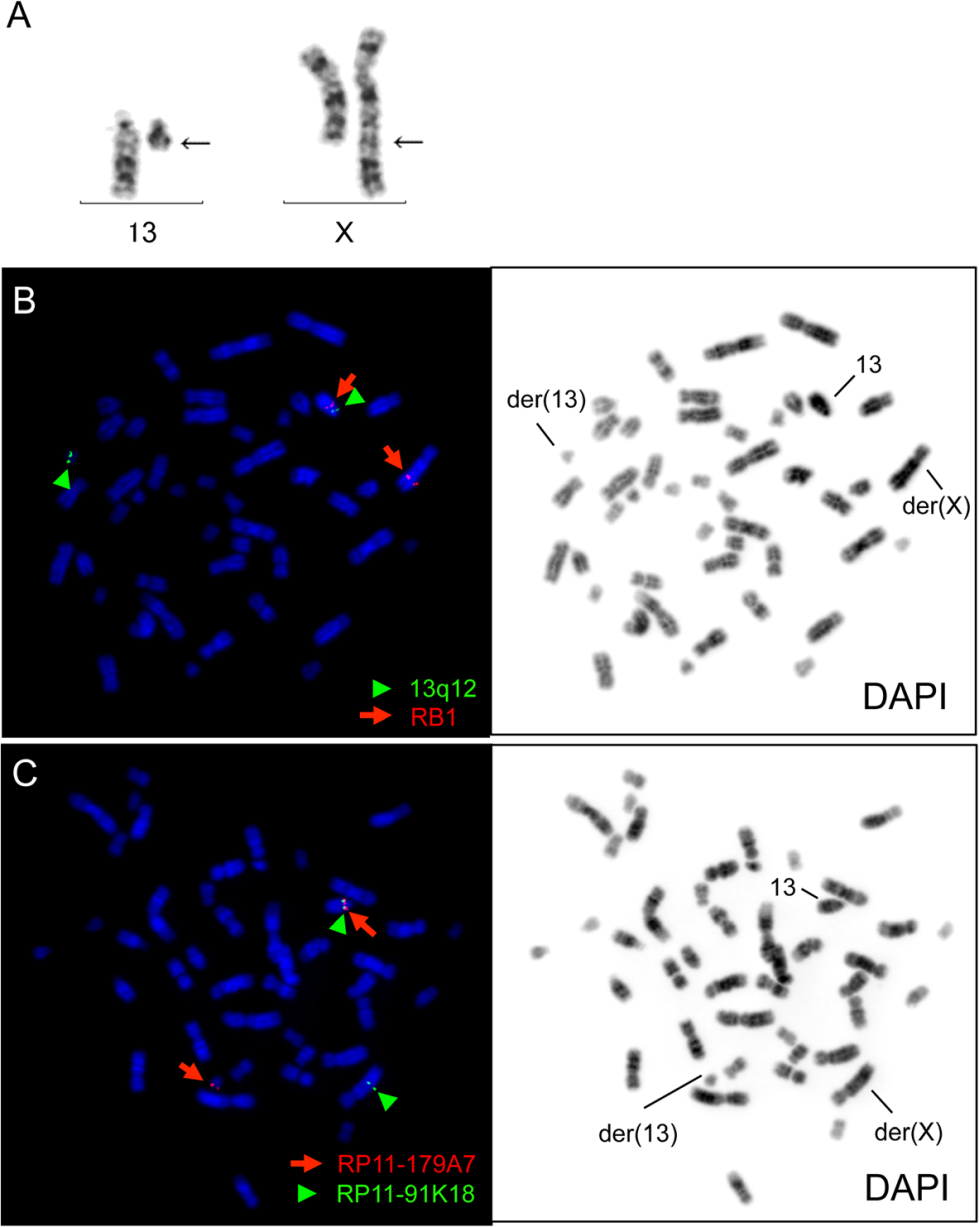
G-banding and FISH analyses of the study patient. (**a**) A G-banded partial karyotype. The arrows indicate the breakpoints of the derivative chromosomes. (**b**) FISH analysis of the *RB1* gene. The arrows and arrowheads indicate *RB1* and 13q12 probes, respectively. (**c**) FISH analysis of the breakpoint on chromosome 13. The arrows and arrowheads indicate RP11-179A7 and RP11-91 K18 probes, respectively

The current study patient was a Japanese girl born at full term with a length of 50 cm and birth weight of 2894 g. G-banding analysis was performed because of her inadequate weight gain at 1 month of age and revealed a de novo balanced reciprocal translocation, t(X;13)(q28;q14.1) (Fig. 1a). She achieved head control at 6 months of age, began to sit up at 10 months, to pull up to a standing position at 12 months, and to walk at 30 months. At 18 months of age, her body length was 74.3 cm (− 1.9 SD), and her weight was 8.3 kg (− 1.6 SD). She was diagnosed with a unilateral retinoblastoma in the left eye (International Intraocular Retinoblastoma Classification, Group D) at 18 months of age. She was then treated with 4 cycles of systemic chemotherapy (vincristine, etoposide, and carboplatin). She suffered from chemotherapy-induced constipation during that period.

The parents refused consent for enucleation of the patient’s left eye although her response to the chemotherapy was found to be inadequate, and side effects such as a tubular disorder were observed. We thus planned for an intra-arterial chemotherapy regimen due to the parents’ wishes. New lesions were developed in the right eye four months later however while waiting for the intra-arterial chemotherapy. Three cycles of intra-arterial chemotherapy for the left eye and various cycles of laser transpupillary thermotherapy (4 cycles for left eye and 2 cycles for right eye) managed to control both eyes and maintain remission for 18 months. However, the retinoblastoma eventually relapsed in the left eye and this was followed by enucleation. The patient was still not talking at 6 years of age, and was thus manifesting severe speech, language and developmental disorders.

#### Breakpoint analysis of chromosome 13

To examine the underlying causes of the phenotype that manifested in our study patient, we analyzed the *RB1* gene by FISH because the chromosome 13 breakpoint was found to be located close to this gene locus at the G-banding level (Fig. 1a). *RB1* signals were detected on the normal chromosome 13 and on der(X), indicating no breakpoint in the *RB1* gene (Fig. 1b). Further FISH analysis with BAC clones mapped the breakpoint to between RP11-179A7 (13q13.2) and RP11-91 K18 (13q13.3), which was 12 to 15 Mb upstream of the *RB1* locus (Fig. 1c). These results indicated that the translocation in our patient did not disrupt the *RB1* gene or its regulatory region. Whole genomic microarray analysis and sequencing of the coding regions of the *RB1* gene revealed no copy number changes or nucleotide variations (data not shown). Thus, we could not map the precise location of the translocation breakpoint using microarray.

#### XCI patterns

**Fig. 2:**
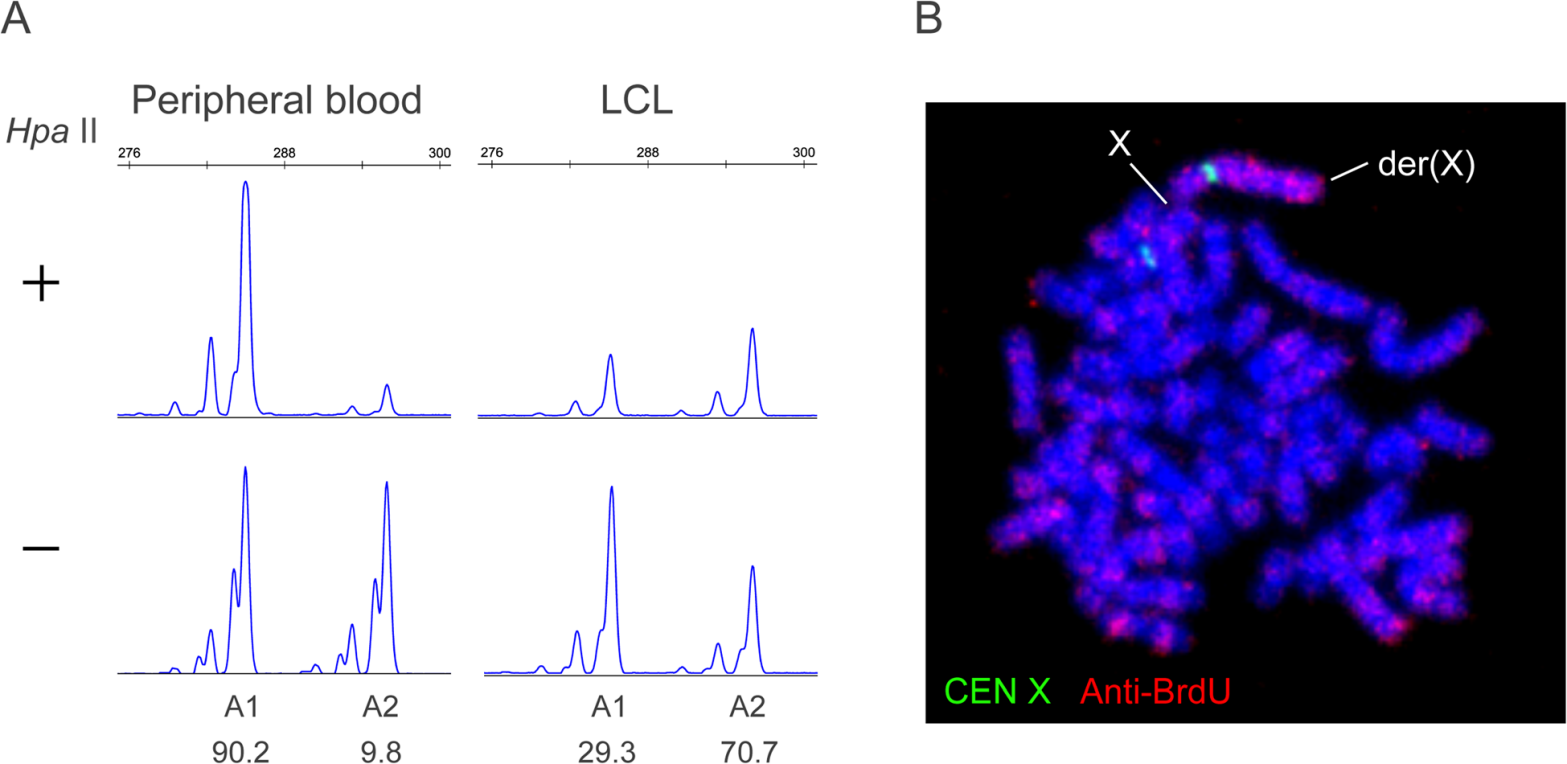
XCI patterns in the peripheral blood and LCL of the study patient. (**a**) HUMARA assay. A1 and A2 represent allele-1 and allele-2, respectively. The percent inactivation of each allele is indicated at the bottom. (**b**) Representative image of a der(X)-inactivated cell. Cells were labeled with anti-BrdU antibody (red) and a centromere X probe (green)

We next assessed whether the der(X) region had been subjected to XCI, which could inactivate *RB1* and nearby genes leading to the retinoblastoma and other symptoms observed in the patient. A HUMARA assay was performed using genomic DNA extracted from peripheral blood. The XCI of allele-1 and -2 was 90.2 and 9.8%, respectively (Fig. 2a). To determine which alleles of the *androgen receptor* gene were located on der(X), we carried out BrdU labeling of the late-replicating heterochromatin in an EBV-transformed LCL (Fig. 2b). Thirty-eight percent of the cells were BrdU-positive at the normal X, whereas the der(X) was positive in 62% of the cells. The XCI of allele-1 and -2 was 29.3 and 70.7%, respectively, in a HUMARA assay of the LCL (Fig. 2a). From these results, we considered allele-1 to be linked to the normal X chromosome, indicating that the XCI was skewed to the normal X in the peripheral blood of our patient.

#### Methylation of the *RB1* gene and other regions of 13q

**Fig. 3:**
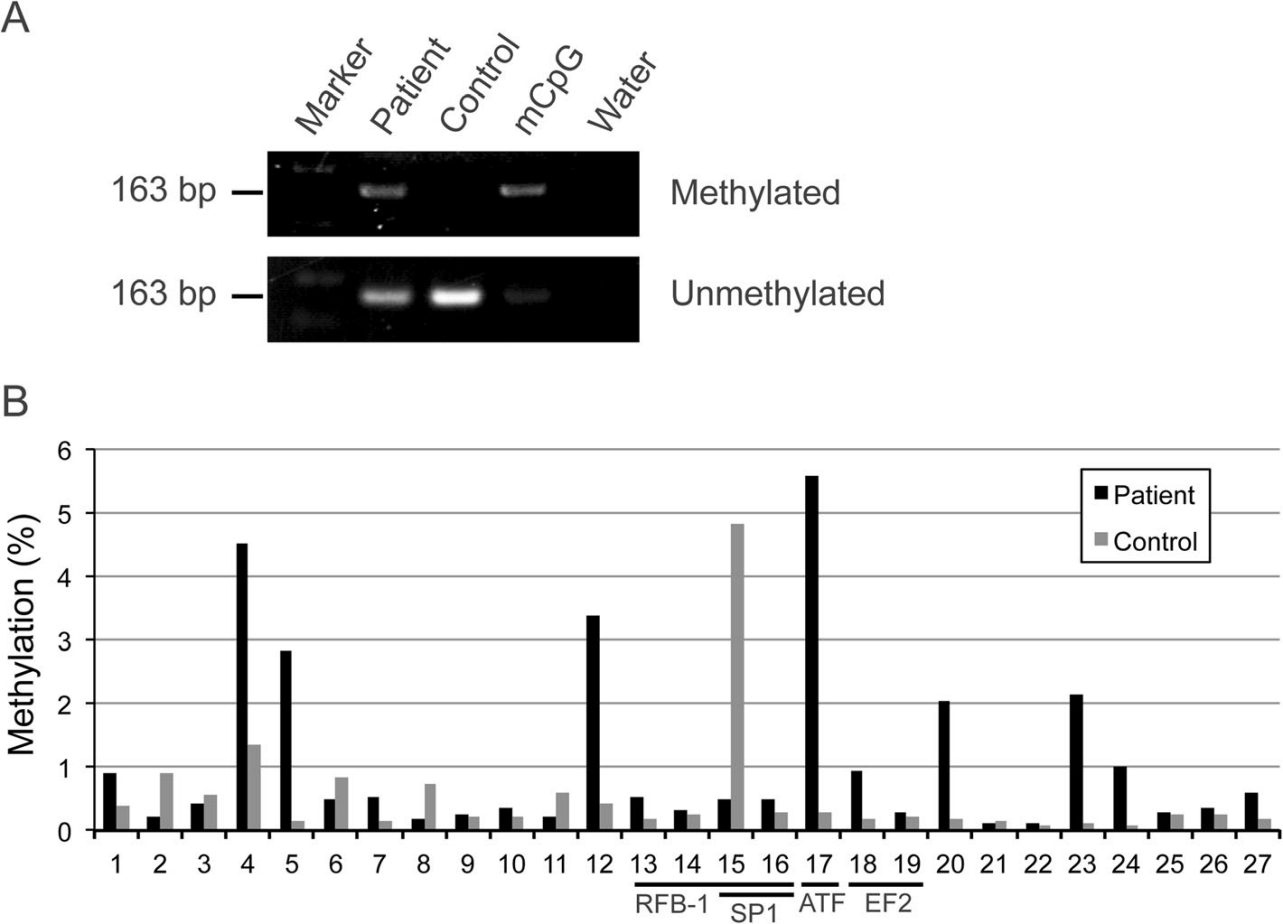
Methylation analysis of the *RB1* promoter in the study patient using bisulfite-treated DNA derived from peripheral blood. (**a**) Agarose gel electrophoresis of MSP products. Amplified products of methylated and unmethylated DNA are indicated. CpG methylated HeLa genomic DNA was used as a positive control (mCpG). (**b**) Frequency of methylation in the 27 CpG sites obtained by bisulfite sequencing; 3.0 × 10^4^ and 1.2 × 10^4^ of next-generation sequencing reads were mapped to each CpG in the patient and healthy control, respectively. The CpGs located within transcription factor binding sites are underlined. Position #15 is a common methylation site

To examine whether the *RB1* gene itself was inactivated in our patient, MSP was performed for the *RB1* promoter using bisulfite-treated DNA as the template. PCR products were detected in the patient and in a positive control but not in a healthy control when a primer pair for amplifying methylated DNA was used (Fig. 3a). The methylation level of the 27 CpG sites in the *RB1* promoter was then investigated using a deep sequencing approach [11]. The patient had a higher methylation frequency than a healthy control (Fig. 3b), with the highest frequency found to be 5.6% at position #17 in her peripheral blood. Given our findings with the HUMARA assay in which ~ 10% of the cells showed the der(X) inactivation (Fig. 2a), we speculated that one *RB1* allele in each cell might be inactivated. Since position #17 is the activating transcription factor (ATF) binding site, methylation of this site might inhibit the binding of transcription factors [12].

We next demarcated the 13q region of inactivation on the der(X) using MSP (Table 2). The region proximal to the breakpoint was not found to be methylated, whereas those distal to it were extensively methylated in our study patient. Although methylation was also detected in regions distal to the *RB1* gene, those of 13q31 were not specific to the patient. Regions near to the 13q terminal were not methylated in the patient.

**Table 2:** MSP amplification of the 13q region in the study patient and healthy controls

	q13.1	q14.11	q14.2 (*RB1*)	q14.3	q21.33	q31.1prox	q31.1 dis	q31.2	q31.3	q32.3	q34
Patient	–	+	+	+	+	+	+	+	+	–	–
Control-1	–	–	–	–	–	+−	+	+−	+	–	–
Control-2	n.d.	n.d.	n.d.	n.d.	–	+	+	n.d.	–	+−	n.d.
Control-3	n.d	n.d.	n.d	n.d.	–	–	+	n.d.	+−	–	n.d.

n.d.: not determined

## Discussion and conclusions

In a similar manner to our present patient, several prior cases of retinoblastoma carrying a constitutional X;13 translocation without disruption of the *RB1* gene had been reported [13–17] and described an inactivation of the derivative chromosome harboring the *RB1* gene [18–23]. The breakpoints of most of these cases including our patient were located at 13q12-q14 regions. To our knowledge, our present case report is the first to demonstrate inactivation of the *RB1* gene at the molecular level i.e. by epigenetic mechanisms. Selective XCI in females with balanced X-A translocations is attributed to a haploinsufficiency of dosage sensitive genes near to the breakpoint in the autosomal region affecting cell viability. Our current case and similar prior retinoblastoma cases harboring X;13 translocations suggest that there are no such critical genes near to the breakpoint on 13q. This would mean that selective XCI of the normal X chromosome in X-A translocation carriers is dependent on the translocation partner chromosome. Moreover, such dosage-sensitive genes may be different between cell lineages, leading to different levels of inactivation among tissues. The HUMARA analysis of the peripheral blood from our current study patient revealed that the normal X was inactivated in 90% of the cells. Although specimens from other tissues in our subject were not available, we speculated that a high frequency of der(X) inactivation would be likely in the retinal cell lineage since our patient suffered from bilateral retinoblastoma. The retinal cell lineage has a relative tolerance to the inactivation of 13q and ironically develop RB. Furthermore, the systemic phenotype of our current study patient other than retinoblastoma implied the presence of a considerable number of cells with an inactivated der(X).

The 13q deletion syndrome is classified into three types depending on the deleted region [5, 24]. Group 1 comprises patients with deletions proximal to 13q32 who show mild or moderate intellectual disability, minor malformations, constipation, growth retardation and inconstant retinoblastoma. Group 2 comprises cases of deletions encompassing 13q32 that show severe intellectual disability, growth retardation, one or more major malformations of the brain, genitourinary and gastrointestinal tract, and distal limb. Group 3 comprises patients with deletions distal to 13q32 who show severe intellectual disability without major malformations or growth retardation. The inactivated region of 13q in our current patient corresponded to group 1 (Table 2), and she had both growth retardation and constipation. However, her intellectual phenotype was more severe than was typically seen in patients categorized as group 1.

**Fig. 4:**
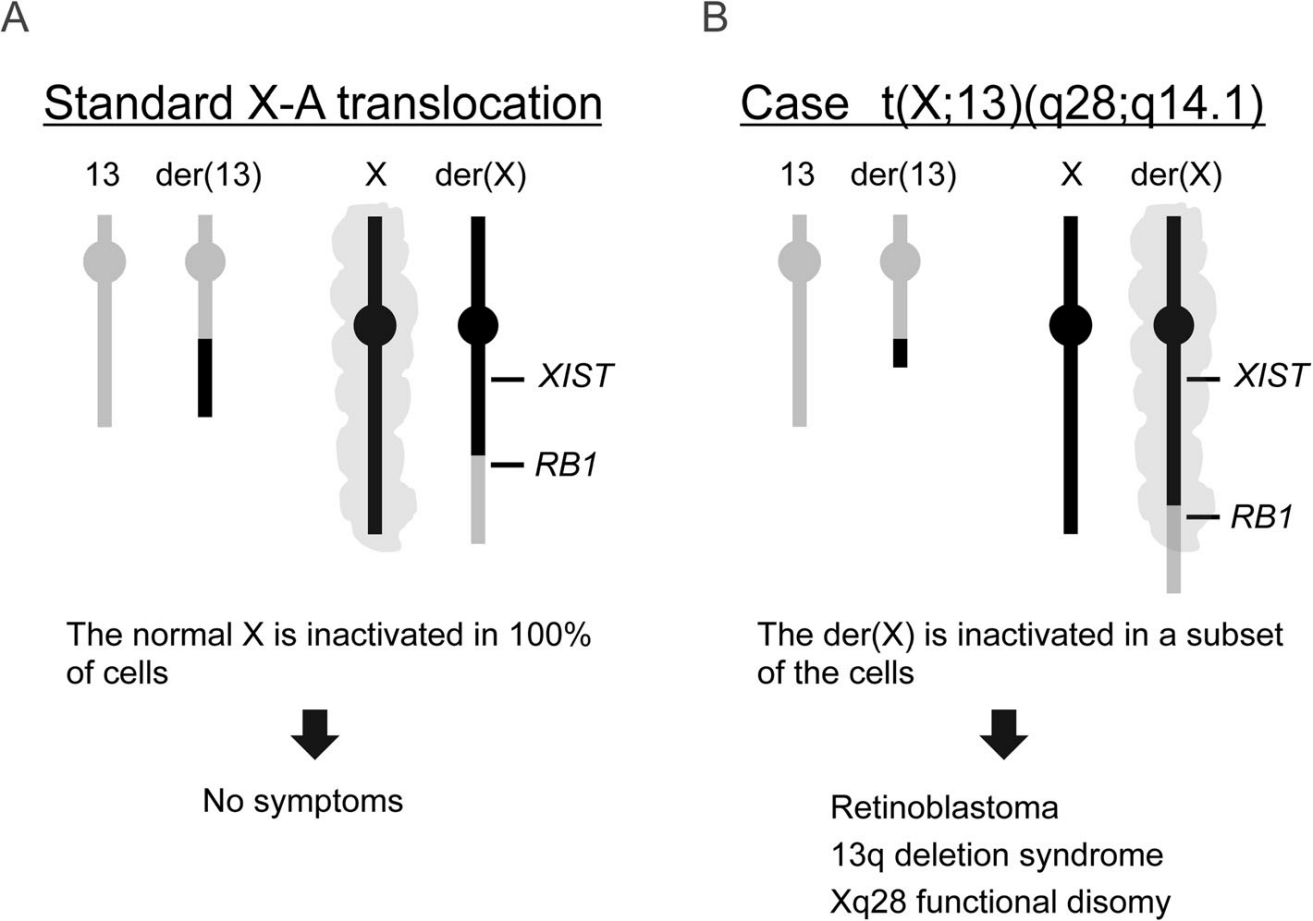
Schematic representation of the XCI pattern and its outcomes with X-A translocation. (**a**) In a standard X-A translocation, the normal X chromosome is inactivated in 100% of the cells because inactivation of the der(X) often leads to suppression of genes indispensable for cell survival. In this situation, the gene dosage is normal and the carrier has no symptoms. (**b**) In the present study case, the der(X) was inactivated in a subset of the cells in which 13q genes including *RB1* on the der(X) were suppressed. This inactivation does not spread to the 13q terminal because of its long distance from the X-inactivation center, allowing the cells to survive. In der(13), the genes located at Xq28 are active. This results in retinoblastoma, 13q deletion syndrome- and an Xq28 functional disomy-like phenotype in such cells

We speculated that the cause of the severe phenotype in our patient originated from a functional disomy of Xq28 which was translocated to der (13). Functional disomy is a situation in which X-linked genes, normally expressed monoallelically, are expressed biallelically in individuals carrying chromosome X-involved structural variants with an unfavorable XCI pattern. As a result, X-linked genes are expressed at a 2-fold higher level than normal [25]. In this case, the der(X) was possibly inactivated in the brain of the patient derived from the common ancestral cell lineage with retina. Thus, Xq28 on the der (13) without the X-inactivation center likely escaped XCI resulting in a functional disomy. Severe developmental delays are common in patients with an Xq28 functional disomy, as was the case in our current patient [26]. The mechanism underlying the onset of retinoblastoma and 13q deletion syndrome- or an Xq28 functional disomy-like phenotype is illustrated in Fig. 4. Our patient was susceptible to the development of retinoblastoma because of the inactivation of the *RB1* gene on the der(X) in her retinal cell linage. A somatic mutation in the other allele on the normal chromosome 13 became the second hit.

We describe a female patient with retinoblastoma and severe intellectual disability, carrying an X;13 translocation. Her *RB1* gene was not disrupted by this translocation but became inactivated by the XCI system. Our current data have important clinical implications. Females carrying an X;13 translocation should be followed-up closely for the early detection of retinoblastoma in infancy and other cancers throughout her life. This should be done even if the XCI is found to be 100% skewed in analysis of peripheral blood samples, because XCI patterns can vary in different tissues. Hence, a female retinoblastoma patient who is a suspected carrier of a germline mutation should be assessed using cytogenetic methods such as G-banding even when conventional analysis reveals no mutations of the *RB1* gene.

## Data Availability

The datasets used and/or analysed during the current study are available from the corresponding author on reasonable request.

## Abbreviations

ATF: Activating transcription factor
BAC: Bacterial artificial chromosome
BrdU: Bromodeoxyuridine
EBV: Epstein-Barr virus
FISH: Fluorescence in situ hybridization
HUMARA: Human androgen receptor assay
LCL: Lymphoblastoid cell line
MSP: Methylation specific PCR
RB: Retinoblastoma
X-A: X;autosome
XCI: X-chromosome inactivation
